# Fluorescence spectroscopy and multispectral imaging for fingerprinting of aflatoxin-B_1_ contaminated (*Zea mays* L.) seeds: a preliminary study

**DOI:** 10.1038/s41598-022-08352-4

**Published:** 2022-03-22

**Authors:** Dragana Bartolić, Dragosav Mutavdžić, Jens Michael Carstensen, Slavica Stanković, Milica Nikolić, Saša Krstović, Ksenija Radotić

**Affiliations:** 1 University of Belgrade, Institute for Multidisciplinary Research, P.O. Box 33, 11030 Belgrade, Serbia; 2 Videometer A/S, Herlev, Denmark; 3 Maize Research Institute, Zemun Polje, Slobodana Bajića 1, 11185 Belgrade, Serbia; 4 Department of Animal Science, Faculty of Agriculture, University of Novi Sad, Novi Sad, Serbia

**Keywords:** Biophysics, Plant sciences, Optics and photonics

## Abstract

Cereal seeds safety may be compromised by the presence of toxic contaminants, such as aflatoxins. Besides being carcinogenic, they have other adverse health effects on humans and animals. In this preliminary study, we used two non-invasive optical techniques, optical fiber fluorescence spectroscopy and multispectral imaging (MSI), for discrimination of maize seeds naturally contaminated with aflatoxin B_1_ (AFB_1_) from the uncontaminated seeds. The AFB_1_-contaminated seeds exhibited a red shift of the emission maximum position compared to the control samples. Using linear discrimination analysis to analyse fluorescence data, classification accuracy of 100% was obtained to discriminate uncontaminated and AFB_1_-contaminated seeds. The MSI analysis combined with a normalized canonical discriminant analysis, provided spectral and spatial patterns of the analysed seeds. The AFB_1_-contaminated seeds showed a 7.9 to 9.6-fold increase in the seed reflectance in the VIS region, and 10.4 and 12.2-fold increase in the NIR spectral region, compared with the uncontaminated seeds. Thus the MSI method classified successfully contaminated from uncontaminated seeds with high accuracy. The results may have an impact on development of spectroscopic non-invasive methods for detection of AFs presence in seeds, providing valuable information for the assessment of seed adulteration in the field of food forensics and food safety.

## Introduction

The most hazardous among aflatoxins (AFs) is aflatoxin B_1_ (AFB_1_), with the highest potential as an environmental carcinogen. The International Agency for Research on Cancer has classified naturally occurring AFs as Group 1 human carcinogens. Currently, more than 5 billion people worldwide suffer from uncontrolled exposure to AFs, and AFs contamination has been linked to increased mortality in farm animals^1^. The diseases caused by AFs consumption are called aflatoxicoses. Chronic aflatoxicosis results in cancer, immune suppression, and other “slow” pathological conditions^1,2^. On the contrary, acute toxicity of aflatoxins has a rapid onset and an obvious toxic response^3^, and leads to death. It has been documented some cases of acute aflatoxicosis in Kenya, India, and Malaysia. The acute lethal dose (LD) for adult humans, children, and most animal species is 10–20 mg, 3 mg, and 0.5–10 mg/kg body weight, respectively^3^. Also, they induce other adverse effects to humans and animals, such as teratogenic, mutagenic, and hepatotoxic effects^3^. They do not only impose severe health risks to humans and livestock, but also lead to huge economic losses lowering the value of grains as an animal feed and as an export commodity^4^. Maize (*Zea mays* L.) is among the most important food commodities for human and animal consumption worldwide^5^ and as such, the condition of maize grains is crucial. With an approximated 25% of the world’s crop being contaminated each year, losses in the billion-dollar range have been estimated, according to Food and Agricultural Organization (FAO) reports^4,6^.

Food contamination is primarily due to naturally occurring contaminants in the environment. The AFs are secondary metabolites produced by molds, particularly by *Aspergillus flavus* and *Aspergillus parasiticus*. Cereal grains, such as maize, are frequently contaminated with AFB_1_. During the pre-harvest stage in the field, the AFs contamination can be more severe; nonetheless, there may be an increase during post-harvest, for example, due to inappropriate storage and transportation^7^. Some works has been reported that AFs have been used as bioweapon agents by Iraq and the Soviet Union^2,8,9^.

Food forensicists need a number of tools to detect the many possible food contaminants^10^ and in this context the detection of AF’s is a prerequisite to insure food safety. As low ppb's concentrations are usually involved, it is important to have very sensitive techniques for their determination. Among them, the most common analytical methods are the enzyme-linked immunosorbent assay (ELISA), high pressure liquid chromatography (HPLC), liquid chromatography coupled to Mass Spectrometry (LC–MS/MS). These techniques are costly, time-consuming and special equipment is required^11^. Hence, optical techniques, including spectroscopy and imaging systems, have been employed for rapid and non-invasive evaluation for the quality and safety of seeds^12^. Fluorescence spectroscopy is a rapid, sensitive, specific and non-invasive technique, which is used for analysis of fluorescence molecules (fluorophores) contained in the samples. Combined with chemometric tools it is widely applied for spectral fingerprints in food analysis^13,14^, such as screening of toxic contaminants, like aflatoxins^15–17^. Multispectral imaging (MSI) technique, provide simultaneously measuring spectral and spatial information of samples (seeds) by imaging their surface reflectance at selected wavelengths^18^. Applications of the MSI for seed analysis have been previously reported by several works^12,18^. Also, an advantage of this technique is the non-invasive and rapid evaluation of the overall quality parameters of the seeds lots, as well as the individual seeds^12^.

In this preliminary study, we applied fluorescence spectroscopy and multispectral imaging on the intact seeds to discriminate maize seeds highly contaminated with AFB_1_ by fungal spores inoculation into plants in field from uncontaminated samples, although there was no any visual difference between these two seed lots. The two optical methods were used to strengthen reliability of the results. Normalized canonical discriminant analysis (nCDA) applied to the multispectral imaging data, and linear discriminant analysis (LDA) applied to the fluorescence spectral data were used to analyse and compare uncontaminated and high-AFB_1_ contaminated seeds. To our knowledge this is first work of this type on the seeds contaminated with AFB_1_ in the natural conditions in field. The results may have an impact on development of spectroscopic non-invasive methods/devices for detection of AFs presence in seeds, which may have practical applications in agriculture and forensics.

## Results

### Fluorescence analysis

**Figure 1:**
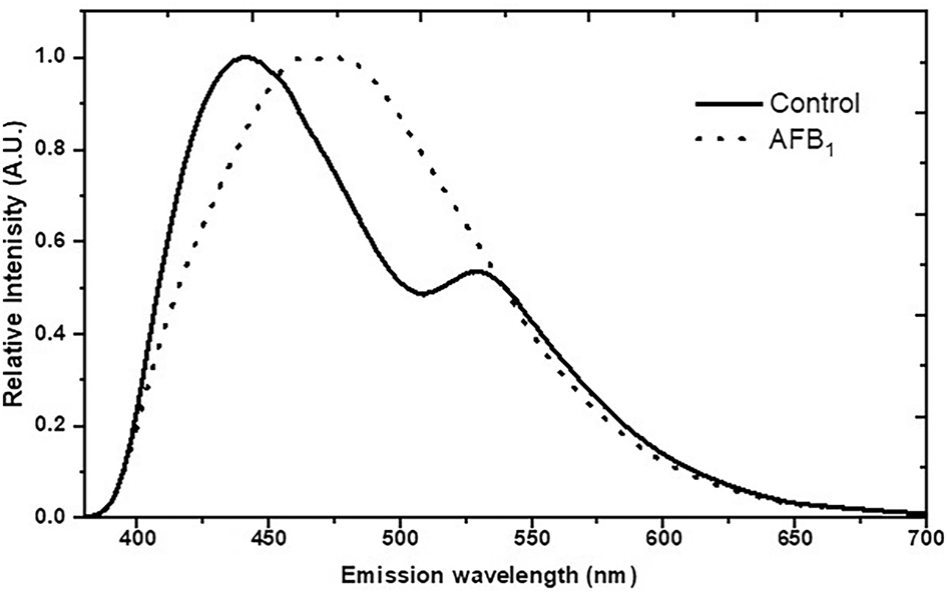
The normalized fluorescence emission spectra shown in solid and dashed lines correspond to control and aflatoxin B_1_-contaminanted *Zea mays* seeds, respectively. Excitation was set at 340 nm.

 Figure 1 illustrates the averaged fluorescence spectra of the control (uncontaminated) and highly AFB_1_-contaminated maize seeds. In the analysed emission region (from 360 to 800 nm) with an excitation wavelength of 340 nm, the differences in the spectral shape and the position of emission maximum were notable. In the control seeds, two emission peaks at 435 nm and 520 nm were observed. By contrast, the high AFB_1_-contaminated seeds exhibited an emission peak at 475 nm, indicating a red shift of the emission maximum position compared to the control samples.

**Figure 2:**
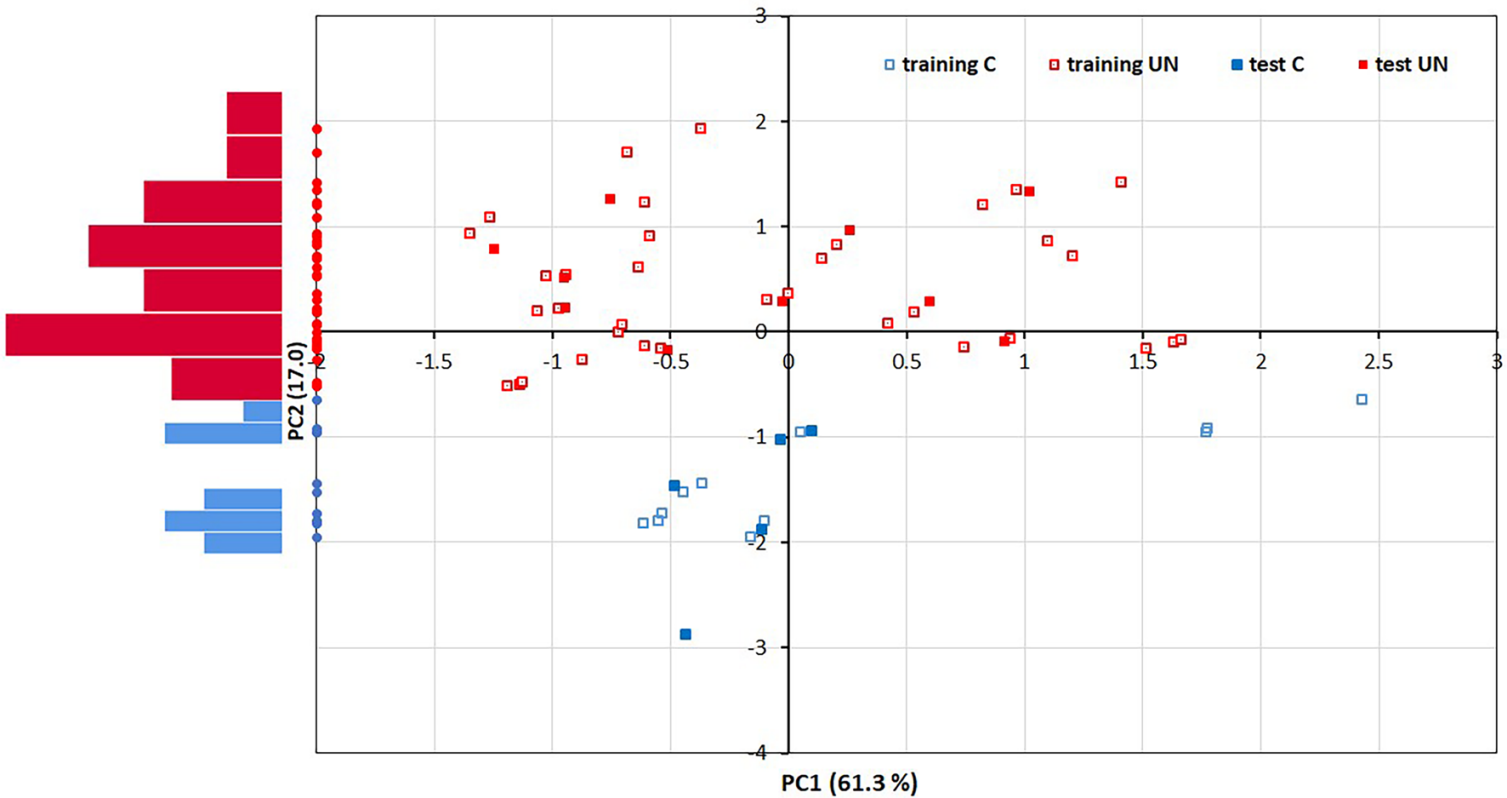
Diagram of scattering of scores corresponding to contaminated and uncontaminated seeds in the space of the first two Principal components. Each score corresponds to one seed. The left side shows the histograms of the scores of the second main component of these two groups.

Linear discriminant analysis (LDA) was performed on scores evaluated by the principal components method. Multicolinearity problem is often present in fluorescence spectroscopy, but this problem was solved by Principal component analysis. This method transforms correlated variables (predictors) into set of uncorrelated variables called Principal components. In this way, multicolinearity problem was solved. In our case, we retained the first two principal components that absorb 78.3% of total variation. The scores of these two principal components were input for linear discriminant analysis. A graphical representation of the scores of the first two principal components is given in Fig. 2, clearly showing the discrimination of the seeds into AFB_1_-contaminated and uncontaminated, as well as the fact that this discrimination was achieved only on the PC2 dimension. The left side of the graph shows the histograms of the PC2 scores for these two groups. The initial data set was divided into a training and a test set in a 46:15 ratio. The results of LDA application are summarized in the confusion matrix (Table 1). Classification reliability of 100% was obtained in both sets.

**Table 1:** Training and test sample confusion matrix for 2-class, AFB_1_-contaminated (C) and control (UN) seeds, classification results.

			Predicted group membership			
			Group	UN	C	Total
Training set^a^	Original	Count	UN	35	0	35
			C	0	11	11
		%	UN	100	0	100
			C	0	100	100
Test set^b^	Original	Count	UN	10	0	10
			C	0	5	5
		%	UN	100	0	100
			C	0	100	100

^a^100% of selected training cases correctly classified.

^b^100% of test original grouped cases correctly classified.

### Multispectral imaging analysis based on reflectance

**Figure 3:**
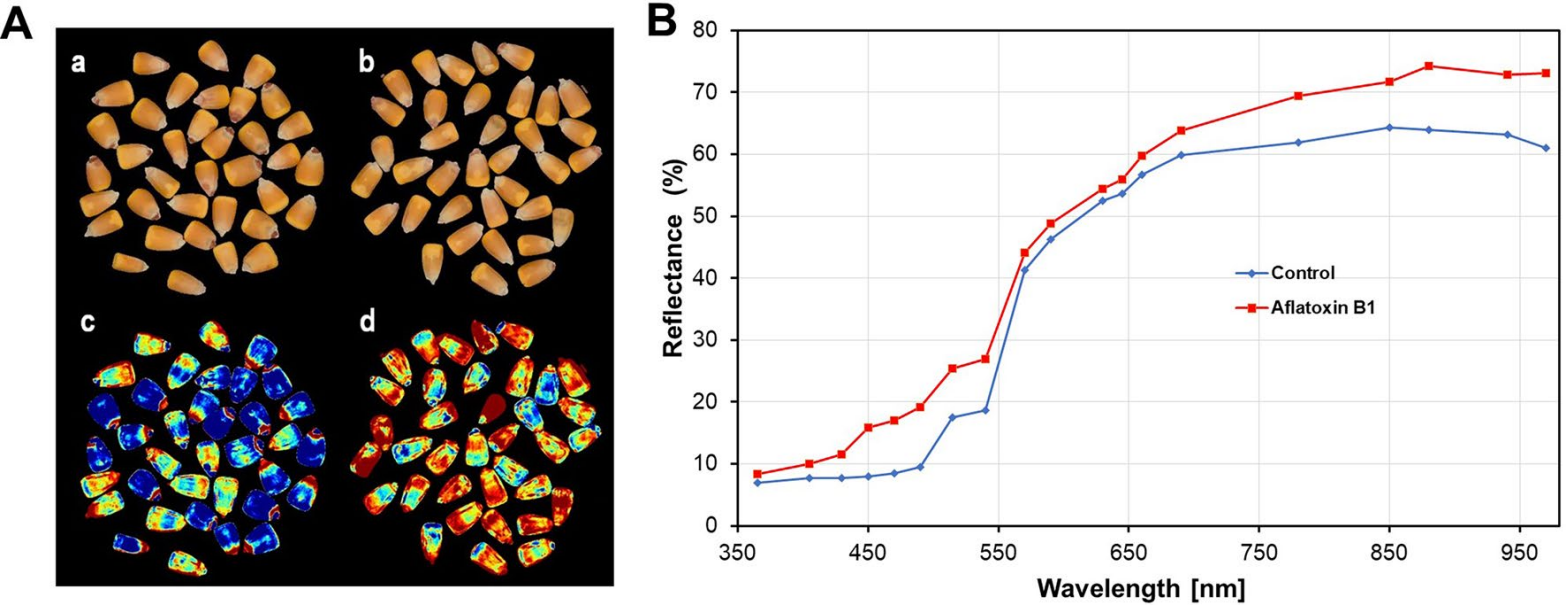
(**A**) sRGB images (a, b) and corresponding nCDA images (c, d) of *Zea mays* L. seedslot for control (uncontaminated) and AFB_1_-contaminated seeds. (**B**) The average reflectance spectra from the multispectral images (**A**) of control and aflatoxin contaminated seeds.

Discrimination between AFB_1_-contaminated and uncontaminated seeds was additionally estimated based on the reflectance intensity in the 375–970 nm region. Figure 3A shows images and corresponding nCDA transformed images of the uncontaminted (a, c) and aflatoxin contaminated (b, d) seeds. Although the analysed seedlots did not differ visually, their nCDA images are showing contrasting differences between the control and the aflatoxin contaminated seeds. We observed that the image of the control seeds displayed more blue pixels, while in the highly AFB_1_-contaminated seeds, there were a more red pixels.

As shown in Fig. 3B, the average reflectance (%) of the control seeds is higher than those of the aflatoxin contaminated seeds in the two spectral regions, from 450 to 540 nm (visible region (VIS)) and from 780 to 970 nm (near infrared region (NIR)).

**Figure 4:**
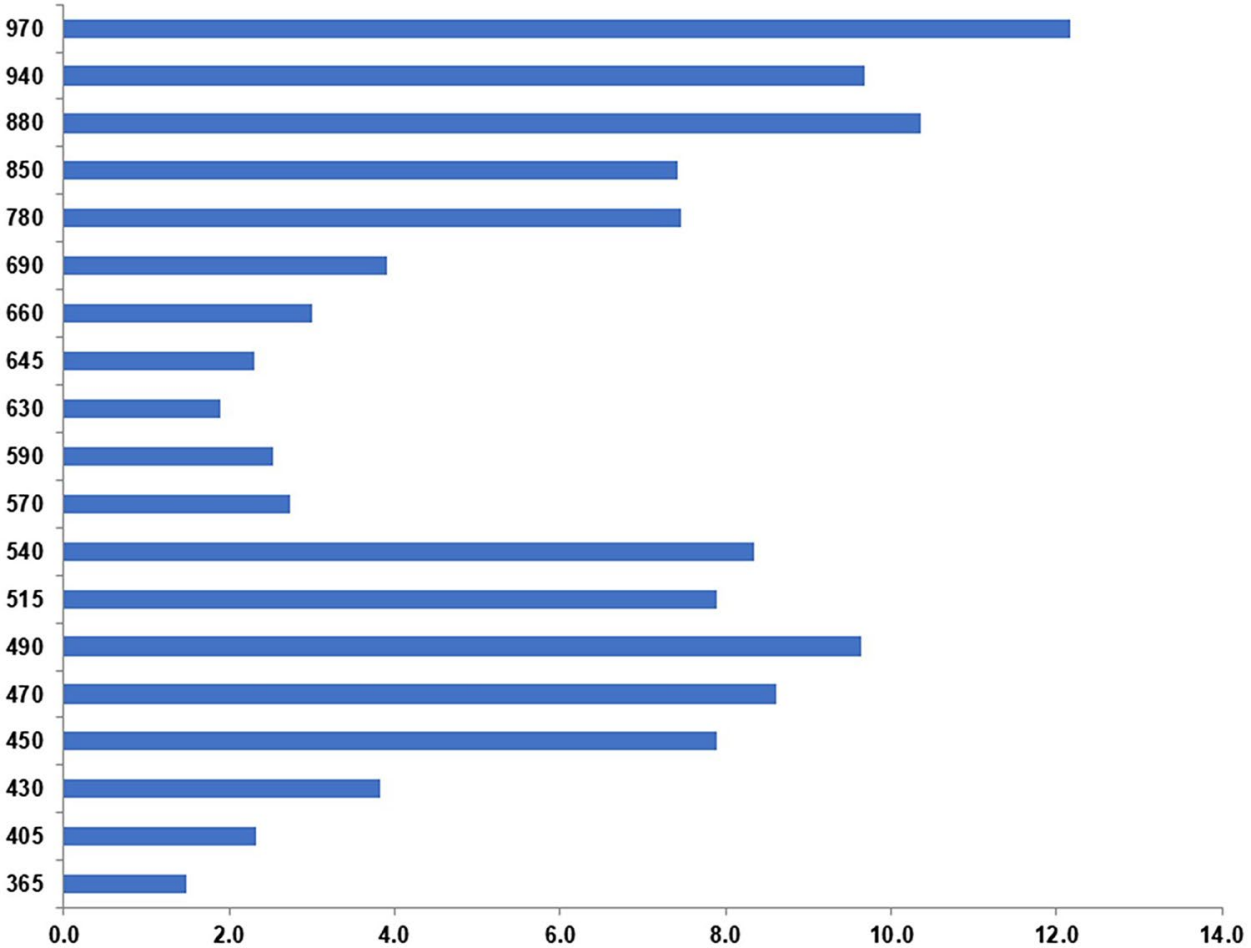
Difference in reflectance intensity between control and contaminated seed samples in the range 350–970 nm.

As shown in Fig. 4, the AFB_1_-contaminated seeds showed a 7.9 to 9.6-fold increase in the seed reflectance for some wavelengths in the VIS region. Our results show an average increase of the reflectance by a factor of approximately 10.4 and 12.2 at 880 nm and 970 nm respectively in aflatoxin contaminated seeds compared to the control samples.

## Discussion

It was previously shown that the positions of the emission maxima were red-shifted in the AFB_1_-contaminated maize flour^15^. These wavelengths correspond to the emission maxima of the various fluorophores, which are mainly found in the plant cell wall, such as polyphenols/lignin^21^. It has been shown previously that some of the phenolic, as well as phenylamide compounds, participate in the seeds’ defense response^22^, such as lignification of the maize seed coat^23^. Also, the carotenoid emit fluorescence in the same spectral region^21^. Our results imply that some of these fluorophores changed in aflatoxin contaminated seeds, which are responsible for the fluorescence characteristics. Linear discrimination analysis (LDA) of the fluorescence data obtained classification accuracy of 100%, to discriminate control and AFB_1_-containing seeds (Fig. 2). However, a more complete assessment requires further studies to be carried out with a higher number of samples.

The difference in reflectance in the visible region between contaminated and control seeds may be due to the change in pigment (e.g. carotenoid) content as a response to contamination^24^. The changes in these pigments content cause change in reflectance intensity. The increase in reflectance of contaminated seeds may be related to the changes in some of the pigments in the seed coat. According to Suwarno et al., the carotenoids content affected the aflatoxin contamination of the grain^25^. Moreover, the colour changes in fungal infected cereal grains could be related to the spectral differences in the VIS region^26^. Also, the structural properties of the surface tissue, in the present case seed coat, may affect reflectance intensity^27^. The thickness of the reflecting layer may also affect reflectance intensity, the higher thickness causing a reflectance intensity increase^28,29^. The thickness of maize seed coat may change as a consequence of the structural changes in the seed coat due to the AFB_1_-contamination. The most important wavelengths in the NIR region were 780, 850, 880, 940 and 970 nm, for both the discriminate aflatoxin and the control seeds. The same spectral wavelengths were also reported in the study of Bianchini et al., which were used to predict the seed quality attributes of *Jatropha curcas* L.^30^. The distinctive spectral patterns correspond to the energy absorption of functional groups containing a hydrogen atom (combination of C–H, N–H and O–H), in the NIR region. Such as, the wavelengths at 890 nm and 940 nm are associated with fat, and fatty tissues are less reflective. The 970 nm wavelength is associtaed with water^31^. The reflectance data also depended on the color in which the brightest regions are the most reflective^30^. It has been reported that unhealthy tissues, such as non-viable seeds, are not good absorbers of NIR energy and have higher reflectance values^30,32^. The observed reflectance changes in contaminated maize seeds could be explained by the changes in the physicochemical properties and pigment content arisen in the AFB_1_-contaminated seeds. In this preliminary study we discriminated highly AFB_1_-contaminated maize seeds from uncontaminated ones. However, in the future work we plan to include various AFB_1_ concentrations in the study, to see the concentration threshold in the application of these methods in discrimination of contaminated from uncontaminated seeds.

In a previous study, fiber optic-fluorescence spectroscopy was used to discriminate artificially contaminated pistachio (*Pistacia vera* L.) kernels with AFB_1_ from uncontaminated samples^16^. In our study, two spectroscopic methods were applied, to our knowledge for the first time on the seeds contaminated with AFB_1_ in field conditions, from uncontaminated seed samples. However, these methods can be applied equally on the seeds naturally or artificially contaminated in the postharvest period.

## Conclusion

By applying fluorescence spectroscopy and multispectral imaging on the intact seeds we discriminated maize seeds highly contaminated with AFB_1_ in field from uncontaminated samples, although there was no any visual difference between these two seed lots. Data analysis was performed using normalized canonical discriminant analysis (nCDA) applied to the multispectral imaging data and linear discriminant analysis (LDA) applied to the fluorescence spectral data. The obtained seeds’ spectral fingerprinting (seeds’ physiological state) could be used to detect poisoned food, and may provide valuable information for the assessment of seed adulteration in the field of food forensics and food safety. We expect that such indicators may be used in forensics for non-invasive and rapid monitoring of unique fingerprint profiles of AFB_1_ in cereal food.

## Materials and methods

### Samples

The samples of maize (*Zea mays* L.) seeds were provided by the Maize Research Institute „Zemun Polje“ (Belgrade, Serbia, harvested in 2019). The use of plants in the present study complies with international, national and/or institutional guidelines. A selected group of hybrids was inoculated by the injection of fungal spore suspension into the silk channel. A method developed by Reid et al*.* (1996) was used for artificial inoculations^19^. Inoculation was carried out 3 days after 50% of plants reached the silking stage. Per cob, 2 ml of inoculum was injected through the silk channel. Five cobs in four replicates were inoculated with such prepared conidial suspension. After harvest, ears were rated for Aspergillus rot and evaluated for levels of aflatoxin contamination. The aflatoxin B_1_ (AFB_1_) content in seeds were determined using the modified AOAC method 980.20^20^. Control (aflatoxin-free) and high AFB_1_-contaminated (1475 µg kg^−1^) seeds were used in this preliminary study.

### Fluorescence measurments using an optical fiber

Fluorescence measurements were performed by an FL3-221 spectrofluorimeter (Jobin Yvon Horiba, Paris, France), equipped with a 450 W high-pressure xenon lamp and a photomultiplier tube. The data were processed using FluorEssence 3.5 software (Horiba Scientific, Kyoto, Japan). The slits on the excitation and emission beams were both fixed at 3 nm. The integration time was 0.1 s. To remove scattering effects, the Rayleigh masking was applied. Fluorescence emission spectra were taken from dorsal surface of the whole seeds, using a quartz optical fibre (4 mm effective diameter). The fluorescence emission spectra of seeds were recorded in the range from 350 to 800 nm, after excitation at 340 nm.

### Multispectral imaging measurments

**Figure 5:**
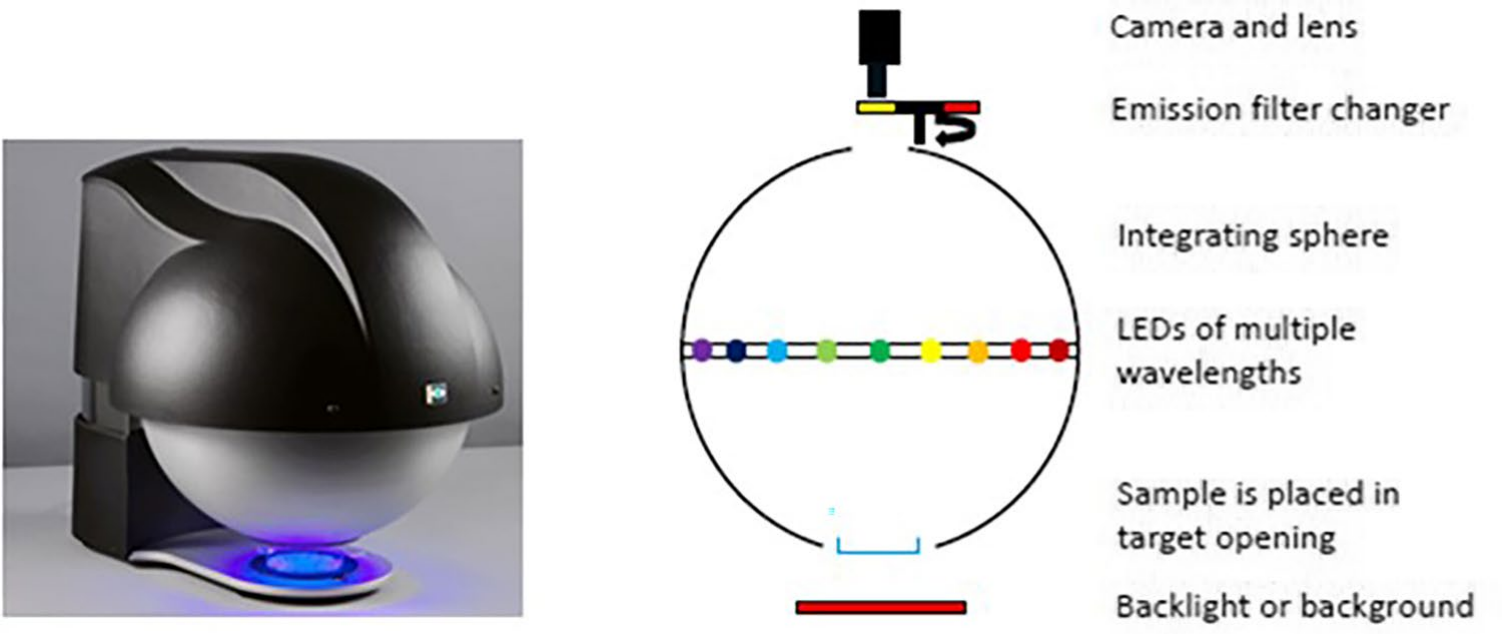
VideometerLab4 instrument (left) and its scheme of the setup for capturing multispectral images (right).

The VideometerLab 4 (Videometer A/S, Herlev, Denmark) device was used for the multispectral imaging analysis of seeds. We randomly selected seeds (around 33), from control and AFB_1_-contaminated seed lot. The seeds were placed on a Petri dish (dorsal surface of seed) for imaging. The multispectral images of 4096 × 3000 pixels, 30 µm pixelsize, FOV, were captured at 19 spectral bands at specific wavelengths from 365 to 970 nm. The picture and scheme of the VideometerLab4 which is used in this study is shown in Fig. 5.

### Permission to collect maize (*Zea mays* L.) seeds

Dr. Slavica Stanković and Dr. Milica Nikolić are employees of the Maize Research Institute, Zemun Polje, Serbia. The seeds are examined on a daily basis at the Institute concerning their disease resistance. The results have been published in numerous scientific journals. Since it is owned by the Institute for which they work, there is not need to ask for a permit.

## Data analysis

### Linear discrimination analysis (LDA)

LDA was applied on the whole fluorescence spectra to study the discrimination of seeds into AFB_1_-contaminated and uncontaminated. The samples were divided into two groups. The first group had 46 samples (35 uncontaminated and 11 contaminated) and was used to train classifiers. The second group was used for testing classifier and had 15 samples (10 uncontaminated and 5 contaminated). For classification of seeds into one of the existing groups, uncontaminated and AFB_1_ high-contaminated, linear discrimination analysis (LDA) was used on scores obtained by the principal components method (PCA). Linear discriminant analysis is a supervised classification method, which can be used to define classification rules into predefined classes. The rules defined in this way can be used to classify new samples, samples whose class affiliation is unknown. In addition, the LDA can be useful in providing information on which variables have the largest contribution to the separation of objects into predefined groups. Technically, the main goal of discriminant analysis is to form linear combinations of independent variables, which enable discrimination between predefined groups, with minimizing the probability of misclassification. This minimisation implies maximizing the relative ratio of variance between and within groups. As the condition for the application of LDA is that the number of objects (samples) be greater than the number of variables, which in this case was not met, it was necessary to reduce the dimensionality of the vector space. This reduction was performed by the principal components method, and the projections of the samples onto the reduced vector space (scores) were used as input for the LDA. In the matrix representation, the model with a given number of principal components has the following form: $\mathbf{X}=\mathbf{T}{\mathbf{P}}^{T}+\mathbf{E}$; $\mathbf{X}$ is the matrix of centered data, $\mathbf{T}$ is the matrix of scores, $\mathbf{P}$ is the lodings matrix, and $\mathbf{E}$ is the error matrix. A linear canonical discriminant function: ${y}_{i}={\mathbf{a}}^{T}{{\varvec{t}}}_{i}$, where ${y}_{i}$ is the discriminant score of the *i*-th sample, and $\mathbf{a}$ is the vector of the linear combination coefficients chosen to maximize the relative ratio between inter and within group variations, while ${{\varvec{t}}}_{i}$ is the score vector of the retained principal components of the *i*-th sample. A cross-validation method was used to assess the accuracy of LDA prediction and involved dividing the data set into a training set and a test set. We used IBM SPSS Statistics for Windows, Version 25.0. (Armonk, NY: IBM Corp. 2017.) for data analysis.

### Normalized canonical discriminant analysis (nCDA)

To discriminate between uncontaminated and aflatoxin contaminated seeds the multispectral images were analysed using a normalized canonical discriminant analysis (nCDA), as described in Olesen et al. (2015) using the built-in software tools of VideometerLab.
